# Electroconvulsive therapy (ECT) in the management of a bipolar disorder with comorbid OCD: a case report and review of the literature

**DOI:** 10.1192/j.eurpsy.2022.1181

**Published:** 2022-09-01

**Authors:** A. Adouni, F. Ben Othman, C. Bencheikh, A. Oumaya

**Affiliations:** The Principal Military Hospital of Instruction of Tunis, Psychiatry, tunis, Tunisia

**Keywords:** bipolar disorder, ECT, OCD, comorbidity

## Abstract

**Introduction:**

Numerous clinical and epidemiological studies show an increased prevalence of anxiety disorders, including obsessive-compulsive disorder, in subjects followed for bipolar disorder. This comorbidity is not without consequence on the evolutionary course of the mood disorder, with frequent relapse, a lower pharmacological sensitivity and an increased risk of suicide.

**Objectives:**

We aim to analyze through a clinical observation and according to the data of the literature the place of ECT in the management of a bipolar disorder with comorbid OCD.

**Methods:**

In this work, we reported a case of a patient in which depressive episodes of bipolar disorder and OCD comorbidity were present and the symptoms were resolved using ECT.

**Results:**

Mrs. TN with family history of suicide had experienced her first depressive episode in 2005 in post-partum, when she had 31 years old. The diagnosis of bipolar disorder II with comorbid OCD was retained after she has had several depressive relapses with few hypomanic ones and reported obsessive suicidal ideation with many religious compulsions. Despite several pharmacotherapeutic combinations (Teralithe, Olanzapine and Venlafaxine) a remission of the symptoms could only be obtained after 15 sessions of ECT that she has had in 2017.

**Conclusions:**

ECT can be a very good substitute for the treatment of mood disorders with comorbid OCD resistant to pharmacotherapy.

**Disclosure:**

No significant relationships.

